# Effects of Low Energy Availability on Bone Health in Endurance Athletes and High-Impact Exercise as A Potential Countermeasure: A Narrative Review

**DOI:** 10.1007/s40279-020-01396-4

**Published:** 2020-12-21

**Authors:** Mark J. Hutson, Emma O’Donnell, Katherine Brooke-Wavell, Craig Sale, Richard C. Blagrove

**Affiliations:** 1 School of Sport, Exercise and Health Sciences, Loughborough University, Loughborough, LE11 3TU UK; 2 Musculoskeletal Physiology Research Group, Sport, Health and Performance Enhancement Research Centre, School of Science and Technology, Nottingham Trent University, Nottingham, NG11 8NS UK

## Abstract

Endurance athletes expend large amounts of energy in prolonged high-intensity exercise and, due to the weight-sensitive nature of most endurance sports, often practice periods of dietary restriction. The Female Athlete Triad and Relative Energy Deficiency in Sport models consider endurance athletes at high-risk for suffering from low energy availability and associated health complications, including an increased chance of bone stress injury. Several studies have examined the effects of low energy availability on various parameters of bone structure and markers of bone (re)modelling; however, there are differences in findings and research methods and critical summaries are lacking. It is difficult for athletes to reduce energy expenditure or increase energy intake (to restore energy availability) in an environment where performance is a priority. Development of an alternative tool to help protect bone health would be beneficial. High-impact exercise can be highly osteogenic and energy efficient; however, at present, it is rarely utilized to promote bone health in endurance athletes. Therefore, with a view to reducing the prevalence of bone stress injury, the objectives of this review are to evaluate the effects of low energy availability on bone health in endurance athletes and explore whether a high-impact exercise intervention may help to prevent those effects from occurring.

## Key Points

**Table:** 

Many endurance athletes suffer low energy availability because of the time and energy demand of training and the need to achieve (or maintain) a target body weight on a regular basis.
Female weight-bearing endurance athletes with symptoms of low energy availability exhibit lower bone mineral density, total and cross-sectional area, estimated strength and thinner cortices, each of which may contribute to the development of bone stress injury. The effects of low energy availability in male athletes are not yet well described.
High-impact exercise may offer a time and energy efficient solution to counter the effects of low energy availability on bone in situations where restoration of energy availability is difficult.

## Introduction

Endurance exercise is energetically demanding, and it is recommended that athletes replenish their energy stores in a timely fashion to enhance recovery and avoid prolonged periods of low energy availability (LEA) [1, 2]. Energy availability is dietary energy intake minus exercise energy expenditure normalised to lean body mass or fat-free mass and is considered low when there is insufficient energy remaining to support optimum physiological function^1^ [3]. During periods in which planned weight loss is a priority (when working towards “race weight”) it can seem counterintuitive to restore energy availability and athletes with rigorous competitive schedules may accumulate many transient periods of LEA. Furthermore, the time demand of high volumes of endurance training can often make it difficult for athletes to consistently meet their energy demands by limiting their opportunities to consume large meals, resulting in inadvertent LEA [4]. As a result, endurance athletes are at high risk of LEA with approximately 31% of female distance runners, and 25% of males, reported to suffer from this condition during training [3, 5].

It is proposed, in the Relative Energy Deficiency in Sport (RED-S) and Female Athlete Triad models, that LEA compromises bone health in athletes, including endurance athletes [1, 4]. LEA is characterized by the perturbation of several hormones involved in the regulation of bone (re)modelling, including the suppression of estrogen, testosterone, leptin, triiodothyronine, and insulin-like growth factor 1, and an increase in adiponectin [6–10]. During a single year, 3–21% of endurance runners may suffer at least one bone stress injury (stress fracture and/or stress reaction) [11–14]. Male and female endurance athletes with greater “Triad cumulative risk scores” are more likely to have suffered a bone stress injury [15, 16]. LEA also causes menstrual disturbances [17], the most severe being functional hypothalamic amenorrhea (FHA), a reversible cause of ovarian disruption characterized by the absence of menses and chronic estrogen deficiency [4]. The highest prevalence of menstrual disturbance is observed in sports emphasizing leanness and endurance sports such as running [18], where it may be as high as 50–65% [19, 20]. Female athletes with FHA suffer more bone stress injuries, and more miss training days due to injury, compared to their eumenorrheic counterparts [5, 21, 22]. Research suggests that high-impact exercises can be osteogenic despite low energy costs [23, 24]; however, their use as a tool to protect bone health and reduce bone stress injuries in athletes at risk of LEA has not yet been considered. In an environment where restoring energy availability (via an increase in energy intake or a reduction in training volume) may be difficult or not considered a priority, such a tool would be beneficial. Therefore, this review aims to (1) investigate the bone changes associated with LEA and (2) explore whether high-impact exercise could mitigate the bone changes associated with LEA. Given that FHA presents as a symptomatic marker of LEA in female athletes, and that FHA is highly prevalent in runners, the focus of this paper will be on weight bearing endurance athletes (WBEA) with FHA. Where the literature exists, evidence in male WBEA with LEA will be also be discussed.

## Effect of Low Energy Availability on Bone Structure and Strength in Endurance Athletes

### Bone Mineral Density and Microarchitecture

Bone health is often viewed as the ability to withstand loading stress and avoid fracture. Bone mineral density (BMD) accounts for 50–70% of the variation in bone strength and low BMD is a risk factor for bone stress injury in WBEA [13, 25–28]. Readers interested in developing their understanding of the bone measures discussed in this paper are directed to a review by Hart et al. [23]. Dual energy X-ray absorptiometry (DXA) produces a 2D image and measures BMD precisely in g cm^2^, but does not ascertain volumetric density or shape and size [23]. Of all the relevant studies retrieved, 56% show that WBEA with amenorrhea had lower BMD at all measured sites compared with their eumenorrheic counterparts (see Table 1). This includes studies that differentiate based on eumenorrhea or non-specific amenorrhea (amenorrhea not necessarily hypothalamic in origin) and may include individuals that use oral contraceptives to regulate irregular menses and women with organic causes of amenorrhea such as polycystic ovarian syndrome. In summarizing only the studies that differentiate participants based on eumenorrhea or FHA, 100% show lower BMD at all measured sites in WBEA with FHA [21, 29, 30]. This suggests that LEA is a critical factor as non-specific amenorrhea is not necessarily attributed to LEA. Longitudinal studies did not show a statistically significant change in BMD over 12 months between FHA and eumenorrheic WBEA [29, 31]. However, BMD was lower at the hip and femoral neck in WBEA with FHA both before and after the 12 month assessment period [29]. It was postulated that most of the BMD loss had occurred prior to entry into the study and a longer assessment period may have been required to capture further loss because the athletes were approaching peak bone mass, where the rate of mineralization slows [32].

**Table 1:** Comparisons of areal bone mineral density, measured at multiple sites using dual energy x-ray absorptiometry, in weight bearing endurance athletes grouped (low vs normal) based on markers of energy availability

Participants	Design	Groups (low vs normal)	aBMD site and direction of group difference when comparing low to normal					Study
			WB ↓	TH ↓	FN ↓	LS ↓	Legs/femur ↓	
129 female weight bearing endurance athletes	Cross-sectional	FHA vs EUM	N/A	+	+	+	N/A	[21]
135 female weight bearing endurance athletes	Cross-sectional	FHA vs EUM	+	+	+	+	N/A	[22]
56 female weight bearing endurance athletes	Cross-sectional and longitudinal	FHA vs EUM	+	+	+	+^b^	N/A	[29]
68 elite female distance runners or triathletes	Cross-sectional	NSA vs EUM	N/A	N/A	+	−	N/A	[34]
34 female weight bearing endurance athletes	Cross-sectional	NSA vs EUM	N/A	+	+	+	N/A	[35]
44 elite female distance runners	Cross-sectional and longitudinal	NSA vs EUM	−	N/A	−	−	N/A	[31]
34 female weight bearing endurance athletes	Cross-sectional	NSA vs EUM	N/A	+	N/A	+	N/A	[30]
29 elite female middle-distance runners	Cross-sectional	NSA vs EUM	+^a^	+^a^	N/A	+	+^a^	[36]
35 elite female endurance race walkers and runners	Cross-sectional	NSA vs EUM	+	N/A	N/A	+	−	[5]
		< 30kcal vs > 30kcal	−	N/A	N/A	−	−	[5]
24 elite male endurance race walkers and runners	Cross-sectional	Low vs normal TES	−	N/A	N/A	−	−	[5]
		< 30kcal vs > 30kcal	−	N/A	N/A	−	−	[5]
40 male and female weight-bearing endurance athletes	Cross-sectional	< 30kcal vs > 30kcal	−	−	N/A	−	N/A	[19]

*aBMD* Areal bone mineral density, *EA* Energy availability, *EUM* Eumenorrhea, *FHA* Functional hypothalamic amenorrhea, *FN* Femoral neck, *H* Total hip, *kcal* kcal⋅kg lean body mass^−1^⋅day^−1^, *LS* L1-L4 or L2-L4 lumbar spine, *N/A* Not applicable, *NSA* Non-specific amenorrhea (may include oral contraceptive users or women with organic causes of amenorrhea), *TES* Testosterone, *WB* Whole body

+ Findings support the corresponding group difference with statistical significance at *p* < 0.05 (unless stated otherwise), − findings do not support the corresponding group difference (*p* > 0.05)

^a^Non-significant trend or statistical analysis reported versus non-athletes only

^b^Significant at 12-months but not at baseline

In contrast, elite male and female runners and race walkers with a calculated seven-day energy availability < 30kcal·kg lean body mass^−1^·day^−1^ (kcal·kgLBM^−1^·day) exhibit similar BMD at the lumbar spine and femur compared to those > 30kcal·kgLBM^−1^·day^−1^ [5]. There are limitations to estimating energy availability via direct calculation, summarized by Burke et al. [33], including the use of an absolute threshold which fails to account for differences between individuals and sex, and the implication that bone health becomes abruptly compromised below a certain value. The physiological responses to 29 and 31kcal·kgLBM^−1^·day^−1^ may be very similar [37, 38]. However, the average energy availability values of the groups above and below 30kcal·kgLBM^−1^·day^−1^ were not reported in the study by Heikura et al. [5] and it could be that the difference between the groups was too small to be physiologically meaningful. Elite male distance runners with testosterone concentrations in the lowest quartile of the group had similar lumbar spine and femoral BMD to counterparts with testosterone concentrations in the other three quartiles [5]. In endurance athletes, however, it is not yet clear what constitutes “low” testosterone and reduced testosterone could be a symptom of the exercise hypogonadal male condition (a condition which has hypogonadal effects similar to RED-S but may not be caused by LEA) rather than a response to LEA [39, 40]. Athletes participating in sports which emphasize leanness are considered high-risk for LEA and many male athletes participating in such sports exhibit low BMD [41]. In male runners, this was associated with a body mass index ≤ 17.5 kg/m^2^ and the belief that being thinner confers a performance advantage [42]. These findings contributed to the development of RED-S [1] and Male Athlete Triad models [1, 38], which recognize the potential relationship between LEA and BMD in males as well as females. FHA, in association with hypoestrogenism and LEA, is clearly associated with reduced BMD in female WBEA. Preliminary data suggest that a similar relationship may exist in male WBEA [38, 41]; however there is a lack of robust evidence from which to draw a firm conclusion.

Bone strength also depends on the mineralization of cortical and trabecular bone [23]. In a systematic review by Mallinson et al. [43] there was no evidence to suggest that cortical mineral density is associated with bone stress injury in athletes. However, impairment in trabecular bone may contribute to increased risk in female WBEA [43–45]. It is important, therefore, to evaluate the effect of LEA on trabecular bone. This has been measured using 3D images produced by high-resolution peripheral quantitative computed tomography (pQCT) at the tibia—a common site of bone stress injury in runners [27, 28]. One study showed that WBEA with FHA had significantly lower trabecular volumetric BMD and number, and significantly higher trabecular spacing, compared to their eumenorrheic counterparts [35]. A similar observation was made in non-athletic women [46]. However, 80% of studies found no significant differences in trabecular volumetric BMD between WBEA with amenorrhea and eumenorrheic counterparts (see Table 2) and one study found trabecular number and thickness was not significantly different in WBEA with FHA [21]. Some of the inconsistency between studies may be due to the grouping of participants based on FHA or non-specific amenorrhea. However, there are also inconsistencies between studies that group athletes based on FHA [21, 22, 35]. As the evidence is cross-sectional, discrepancies could be due to differences in duration and severity of LEA between samples which was not accounted for. Mitchell et al. [21] measured trabecular microarchitecture using high-resolution pQCT, and showed significantly fewer trabecular plates, thinner trabecular rods, and suboptimal trabecular alignment in WBEA with FHA compared to their eumenorrheic counterparts, which was associated with prior stress fracture. The evidence regarding trabecular mineral density and microarchitecture in WBEA with LEA is either inconsistent or has not yet been replicated and the true effect will remain unknown until randomised controlled trials are conducted; however, some data have shown that it is compromised in female WBEA with LEA and this could be important in terms of injury risk [21].

**Table 2:** Comparisons of trabecular mineral density and microarchitecture at the tibial epiphysis, measured using peripheral quantitative computed tomography, in weight bearing amenorrheic and eumenorrheic endurance athletes in studies using a cross-sectional design

Participants	Groups (low vs normal)	Trabecular mineral density and microarchitecture parameter and direction of group difference when comparing low to normal				Study
		TB vBMD ↓	TB number ↓	TB spacing ↑	TB thickness ↓	
129 female weight bearing endurance athletes	FHA vs EUM	−	−	N/A	−	[21]
135 female weight bearing endurance athletes	FHA vs EUM	+^a^	N/A	N/A	N/A	[22]
34 female weight bearing endurance athletes	FHA vs EUM	+	+	+	−	[35]
34 female weight bearing endurance athletes	NSA vs EUM	+^a^	N/A	N/A	N/A	[30]
29 elite female middle-distance runners	NSA vs EUM	−	N/A	N/A	N/A	[36]

*EA* Energy availability, *EUM* Eumenorrhea, *FHA* Functional hypothalamic amenorrhea, *N/A* Not applicable, *NSA* Non-specific amenorrhea (may include oral contraceptive users or women with organic causes of amenorrhea), *TB* Trabecular, *vBMD* Volumetric bone mineral density

+ Findings supports the corresponding group difference with statistical significance at *p* < 0.05 (unless stated otherwise), − findings do not support the corresponding group difference (*p* > 0.05)

^a^Non-significant trend or statistical analysis reported versus non-athletes only

### Bone Geometry and Estimated Strength

Bone area influences bone strength independent of bone mineral density [23], and total and cortical area have been linked with bone stress injury in athletes [28, 45, 47]. However, 83% of studies that have investigated the effect of LEA at the tibia showed no significant difference in total or cortical area between WBEA with FHA (or non-specific amenorrhea) and eumenorrheic counterparts (see Table 3). One of these studies showed a lower cortical area in 16 WBEA with FHA compared to 18 eumenorrheic controls that was non-significant, but had a large effect size (Cohen’s *d* = 0.75) [35]. Similar, non-significant, group differences in cortical area have also been shown graphically in another study on 19 WBEA with non-specific amenorrhea [36]. These studies were performed on a relatively small group of athletes and the lack of significance may have been due to sample size. Ackerman et al*.* [48] showed that cross-sectional area was significantly lower at the femoral neck, shaft and trochanter in WBEA with FHA compared to eumenorrheic WBEA, whereas Piasecki et al. [36] showed that cross-sectional area at the femoral neck and shaft was no different in participants with non-specific amenorrhea. However, these findings should be interpreted with caution because cross-sectional area could only be estimated by making assumptions based on 2D DXA images.

**Table 3:** Comparisons of bone area and computed simulated compressive strength in weight bearing amenorrheic and eumenorrheic endurance athletes in studies using a cross-sectional design

Participants	Groups (low vs normal)	Measured using	Site	Bone area or simulated strength parameter and direction of group difference when comparing low to normal					Study
				T/CS area ↓	CT area ↓	TB area ↓	Stiffness ↓	Failure load ↓	
79 female weight bearing endurance athletes	FHA vs EUM	DXA	Femoral neck	+	N/A	N/A	N/A	N/A	[48]
			Femoral shaft	+	N/A	N/A	N/A	N/A	[48]
			Trochanter	+	N/A	N/A	N/A	N/A	[48]
68 elite female runners and triathletes	NSA vs EUM	DXA	Femoral neck	+	N/A	N/A	N/A	N/A	[34]
29 elite female runners	NSA vs EUM	DXA	Femoral neck	−	N/A	N/A	N/A	N/A	[36]
			Femoral shaft	−	N/A	N/A	N/A	N/A	[36]
129 female weight bearing endurance athletes	FHA vs EUM	HR-pQCT	Tibial epiphysis	−	N/A	−	N/A	+	[21]
135 female weight bearing endurance athletes	FHA vs EUM	HR-pQCT	Tibial epiphysis	+^b^	−	−	+^a^	+	[22]
34 female weight bearing endurance athletes	FHA vs EUM	HR-pQCT	Tibial epiphysis	−	+^a^	−	N/A	N/A	[35]
34 female weight bearing endurance athletes	FHA vs EUM	HR-pQCT	Tibial epiphysis	−	+^a^	−	+^a^	+^a^	[30]
29 elite female middle-distance runners	NSA vs EUM	pQCT	Tibial epiphysis	−	N/A	−	N/A	N/A	[36]
29 elite female middle-distance runners	NSA vs EUM	pQCT	Tibial diaphysis	−	+^a^	N/A	N/A	N/A	[36]

*CS* Cross-sectional, *CT* Cortical, *DXA* Dual energy x-ray absorptiometry, *EA* Energy availability, *EUM* Eumenorrhea, *FHA* Functional hypothalamic amenorrhea, *HR-pQCT* High-resolution peripheral quantitative computed tomography, *N/A* Not applicable, *NSA* Non-specific amenorrhea (may include oral contraceptive users or women with organic causes of amenorrhea), *pQCT* peripheral quantitative computed tomography, *T* Total, *TB* Trabecular

+ Findings supports the corresponding group difference with statistical significance at *p* < 0.05 (unless stated otherwise), − findings do not support the corresponding group difference (*p* > 0.05)

^a^Non-significant trend or statistical analysis reported versus non-athletes only

^b^Significant when controlled for age of menarche

Cortical thickness is a geometric factor of bone strength, and reduced cortical thickness has been associated with bone stress injury in military recruits [23, 49]. Data from the only study to investigate cortical geometry at the tibial diaphysis showed that endocortical circumference (inner circumference of the cortical layer) was greater in amenorrheic WBEA compared to eumenorrheic counterparts, whilst periosteal circumference (outer circumference of the cortical layer) was no different [36]. It was suggested that this represents enhanced endocortical resorption during LEA, with no concomitant periosteal apposition. This would result in thinner cortices, as was shown at the tibial epiphysis, femoral neck and trochanter and in WBEA with FHA [30, 48]. Although total bone area may not be affected, these findings suggest that LEA may promote cortical thinning (and potentially reduce cortical area) in WBEA, as has been shown in exercising women [46] and military recruits with oligomenorrhea [50].

Five studies have provided at least one bone strength estimate in WBEA with amenorrhea, including section modulus (an index of strength in bending) [34, 48], strength index (the ratio of yield strength to expected force of a fall) [36], buckling ratio (a measure of susceptibility to cortical buckling) [48], cross-sectional moment of inertia (an estimate of resistance to bending) [36, 48], stiffness (resistance to deformation) [22, 30] and failure load [21, 22, 30]. Failure load (mechanical load at failure) can be measured using finite element analysis (computer simulated axial compression of bone) and was significantly lower at the tibial epiphysis in WBEA with FHA compared to eumenorrheic counterparts [21, 22]. No other bone strength estimate showed a statistically significant difference between WBEA with FHA (or non-specific amenorrhea) and eumenorrheic counterparts. The reason for the discrepancy between failure load and other strength estimates is not clear. However, finite element analysis predicted ex vivo load at failure of a cadaver radius under compression (*R*^2^ = 0.66, *p* < 0.001) with greater agreement than BMD (*R*^2^ = 0.31, *p* < 0.001) and the best combination of several bone mineral and geometric parameters used in other strength estimates (*R*^2^ = 0.47, *p* < 0.001) [51]. The agreement between these predictions and actual load at failure is likely to differ depending on the type of loading that is applied to bone (e.g. compression or torsion). Nevertheless, the data support the notion that lower failure load in WBEA with FHA is detrimental to bone strength [21, 22].

There are associations between FHA and impaired BMD, trabecular volumetric BMD, number and spacing, cortical thickness and failure load. This suggests that LEA may lead to weaker bones that are narrower and of lower mass which may, in turn, increase the risk of bone stress injury in WBEA [47]. In agreement, following a systematic review of the literature, Mallinson et al. [43] concluded that cortical thickness and area may play a critical role in the development of bone stress injury in athletes with menstrual dysfunction. However, other dietary changes (e.g. micronutrient status) may accompany a change in energy availability and the contribution of such changes to the effects discussed is not clear. Other dietary factors that may influence bone health are discussed in detail elsewhere [52]. Furthermore, the primary causative factor of FHA in female athletes, energy deficiency, is also associated with chronic estrogen deficiency. Interactions between energy and estrogen deficiency lead to additive decrements to bone [53], yet both may exert independent effects also [46]. Therefore, an athlete suffering from LEA but not estrogen deficiency may experience different (and possibly mitigated) bone changes. Data have been interpreted from studies using a cross-sectional design such that group differences in any other confounding variable may have contributed to the observed findings independent of energy availability, and evidence in male athletes is limited. The lack of longitudinal and controlled trials is likely due to difficulties in accurately monitoring changes in energy availability, and the ethical implications of controlling it, for periods long enough to observe structural bone change. Research must focus on developing and validating markers of LEA to address these gaps in the literature.

## High-Impact Exercise to Promote Bone Health in Endurance Athletes at Risk of Low Energy Availability

Any exercise intervention in trained athletes, suffering from LEA, would require a unique set of characteristics to effectively protect bone from adverse effects. It is crucial that the intervention does not exacerbate the existing energy deficiency as this has been shown to accelerate bone loss [54]. Thus, the energy cost should be as little as possible because it is unlikely that an athlete will succeed in replacing large amounts of calories when their energy availability is already compromised. If the intervention were to be a burden on time, it is unlikely that athletes would be able to integrate the exercise into their training schedules without compromising the volume of their existing training—which is typically high in endurance sports. The Mechanostat theory states that a loading stimulus must exceed a minimum strain threshold to elicit an adaptive response [55]. Such a threshold is likely higher in WBEA (compared to non-athletes and non-weight bearing athletes) due to years spent performing weight-bearing activity, such that particularly high loading rates are required [55]. Therefore, an exercise intervention needs to be brief, cost as little energy as possible, and elicit a high level of strain, to protect the bones of WBEA suffering from LEA.

A high level of strain can be exerted by high magnitude, low frequency loads applied at a fast rate, such as during jumping and bounding, and can be enhanced further still by incorporating multi-directional impacts [23]. Furthermore, mechanosensitivity to such a stimulus dampens after a relatively small number of loading cycles and is only restored after a re-sensitization period of 8–24 h [23, 56, 57]. In theory, performing low repetition high-impact exercise at low frequency (up to three times per day) represents a model whereby a maximum adaptive response can be achieved in a timely manner. This may depend on an athlete’s ability to plan this exercise and allow time between training sessions to minimize interference between the high-impact stimulus and regular training. It is not yet known how long this would need to be. Athletes would expend a negligible number of calories performing high-impact exercise (making it easy to compensate for), however, even if the energy cost is not fully accounted for, it is possible that the high levels of strain could outweigh the effects of a marginally lower energy availability. Most importantly, the effects of the intervention would need to oppose the effects of LEA on bone, and continue to do so during periods of LEA.

## Effect of Low Repetition High-Impact Exercise on Bone Structure and Strength

### Bone Mineral Density and Microarchitecture

There are limited data available regarding the effect of high-impact interventions on bone health in WBEA. However, high-impact jumping interventions (e.g. 20 countermovement jumps weighted with up to 5kg, 3–4 times per day, 3–4 times per week) significantly increased bone mineral content and cross-sectional area at multiple load bearing sites in male adolescent cyclists [58, 59], and men with low bone mass [60]. Similar effects did not occur in adolescent soccer players and it was suggested that the osteogenic benefits of a history of soccer training are such that the minimum strain threshold was higher and the intervention failed to exceed it [58, 59]. Considering the osteogenic benefit of distance running is less than it is in soccer [61], WBEA may increase bone mineral content and cross-sectional area following a similar intervention. In premenopausal women, daily jumping interventions (as few as ten maximum vertical jumps a day, three times a week) also significantly increased femoral neck cross-sectional area, BMD and content [24, 62–65], lumbar spine BMD and content [24], and hip BMD [60, 66]. Due to the difficulties in estimating changes in bone size from 2D DXA images, bone gain or loss may be best interpreted via changes in bone mineral content—which were mostly comparable to changes in BMD and cross-sectional area. The data show that high-impact interventions increase bone mineral density and mass in the absence of LEA.

Suominen et al. [67] showed that there was no significant effect of a 5-month training intervention (including multi-directional plyometric jumping and bounding) on trabecular volumetric BMD at the tibial epiphysis in elite male masters sprinters. The intervention incorporated impact exercises once or twice per week but a greater emphasis was placed on resistance training, which is associated with bone adaptation at the diaphysis rather than at the epiphysis [68]. Furthermore, masters sprinters may have more mineralized bones compared to WBEA such that a greater loading rate is required to stimulate adaptation [69]*.* It was postulated that the intervention used by Suominen et al. [67] provided insufficient impact stimulus to improve trabecular volumetric BMD in masters sprinters*,* although this may not be the case for WBEA completing a high-impact intervention*.* A 10-month multidirectional jumping intervention consisting of two 40–45 min sessions per week had no significant effect on change in trabecular volumetric BMD from baseline in a group of young women; however there was no non-exercise control group [68]. A three-month single leg jumping intervention (10–20 jumps, 3–4 times per day) had no significant effect on trabecular number, spacing or bone volume fraction (representative of trabecular mineralization) compared to the non-exercised leg in postmenopausal women [70]. It was suggested that the 3-month intervention was too brief to elicit a significant change in mineralization [70]. Unfortunately, no data were available on trabecular microarchitecture (structure of trabecular plates and rods) in response to high-impact exercise. The evidence suggests that high-impact interventions do not improve trabecular mineral density or microarchitecture; however, this may be less critical given a number of studies have shown that these parameters are not impaired in WBEA with LEA (see Table 2) or athletes with a history of bone stress injury [43]. Furthermore, the findings are limited, and the effects of a daily intervention lasting longer than 3 months are not yet clear.

Interestingly, it has been shown that trabecular volumetric BMD in WBEA with FHA is significantly impaired at the non-weight bearing radius compared to eumenorrheic counterparts (*p* < 0.05, *d* = 0.78) but not at the weight bearing tibia (*p* > 0.05, *d* = 0.77) [35]. This suggests that tibial loading may have helped mitigate the effects of FHA on trabecular volumetric BMD. However, both effect sizes were moderate implying that there is a degree of individual variability in the response to loading, or that the study lacked statistical power. Furthermore, a 6-month low repetition hopping intervention significantly increased femoral neck BMD in postmenopausal women despite estrogen deficiency [71]; female athletes with FHA also demonstrate estrogen deficiency [72]. In overweight (and otherwise sedentary) individuals, BMD was significantly reduced following 12 months of caloric restriction-induced weight loss but was maintained during weight bearing exercise-induced weight loss of a similar magnitude and duration [73]. These studies suggest that benefits of impact loading could persist during periods of LEA and associated estrogen deficiency.

### Bone Geometry and Estimated Strength

LEA has been associated with reduced cortical thickness and total area in WBEA (see Table 3). Cortical thickness and total and cortical area significantly increased at the tibial diaphysis following a 5-month training intervention in elite male masters sprinters compared to sprint training alone [67]. However, it was not clear whether these benefits were driven by impact or resistance exercise, as the intervention included both. In contrast, the only studies to measure total and cortical area at the tibial epiphysis in response to a high-impact intervention showed no effect [67, 70]. One of the studies (in elite male masters sprinters) may not have provided sufficient impact stimulus to drive adaptation, and the other (a 3-month intervention) was perhaps not long enough [67, 70]. As such, the effect of high-impact interventions on bone area at the tibial epiphysis remains unclear. However, this may not be critical in terms of preventing the effects of LEA as studies have shown that total area is not significantly lower at the tibial epiphysis in WBEA with LEA compared to eumenorrheic counterparts (see Table 3). In response to an intervention including only high-impact exercise, inactive young women also significantly increased cortical thickness at the tibial diaphysis [68]. Furthermore, a 7-month low repetition jumping intervention significantly increased cortical thickness at the femoral neck in early pubertal girls (Tanner stages 2 and 3) compared to normally active non-intervention controls [65]. In both studies, there was no significant change in periosteal circumference and it was suggested that suppressed endocortical resorption contributed to thicker cortices in the women who received the intervention [65, 68]. This effect may differ in men or amenorrheic women due to the effects of estrogen on localized bone gains [74]. However, animal studies support the notion that daily jumping interventions reduce endosteal expansion leading to thicker cortices [75]. This body of research shows that high-impact interventions increase cortical thickness and area at sites where the opposite effect has been associated with LEA and bone stress injury in WBEA.

No study has measured failure load in response to a high-impact intervention; however, athletes participating in high-impact sports exhibit greater estimated failure load at the tibial epiphysis compared to athletes in lower impact sports [76]. In reference to other bone strength estimates, elite male masters sprinters significantly increased minimum cross-sectional second order moment (reflects resistance to bending in the direction of smallest rigidity) at the tibial diaphysis following a high-impact and resistance intervention [67]. Similarly, femoral neck cross-sectional moment of inertia significantly increased in adolescent male swimmers and cyclists, and section modulus increased by ~ 6% in cyclists and by ~ 9% in swimmers, following a 9-month low repetition jumping intervention compared to controls [58]. In the same study, soccer players exhibited a similar ~ 6% increase in section modulus. It is interesting that despite the habitual osteogenic stimulus of soccer training, this group of athletes experienced a quantitatively similar increase in section modulus compared to non-weight bearing endurance athletes [58]. Weight bearing endurance training is not as osteogenic as soccer, and therefore it is likely that WBEA would also improve section modulus following a similar intervention [61]. Femoral neck section modulus also significantly increased in response to high-impact interventions in inactive premenopausal women [64] and early pubertal girls [65]. The only evidence of an association between LEA and estimated bone strength is in terms of failure load estimated under compression. Although resistance to bending at the femoral neck and tibial diaphysis has been shown to improve in response to high-impact interventions, it is not clear whether this would mitigate the effects of LEA or reduce the risk of tibial bone stress injury.

There is evidence to suggest that a high-impact intervention may help mitigate the effects of LEA on bone strength; however, further research needs to be conducted before it could be considered a viable tool to protect athletes suffering LEA from bone stress injury. Many of the interventions studied were unidirectional, which is not the optimum loading pattern for osteogenic stimulus [23], and have only been tested in non-athletes. If WBEA have stronger bones than non-athletes, their response to high-impact interventions may differ [55]. The only data regarding the effects of a high-impact intervention during LEA are from cross-sectional studies or overweight and postmenopausal populations. Also, to address these issues, future research should aim to test the effects of a multi-directional low repetition high-impact intervention during LEA on bone mineral density, geometry and estimated strength using a longitudinal or controlled design in male and female WBEA.

## Effects of Short-Term Low Energy Availability and Impact Exercise on Circulating Markers of Bone (Re)modeling

The use of bone (re)modeling markers to monitor the effects of bone treatment has been supported by the International Osteoporosis Foundation as they respond more rapidly than structural measures such as BMD [77]. To investigate a cause and effect relationship between LEA and bone health, research has sought to control energy availability and measure the acute change in systemic markers of bone (re)modeling. In the only study on WBEA, eight male distance runners exercised for three days in energy balance in one condition, and at 50% of energy balance in another. Significant reductions in serum pro-peptide of type 1 collagen (P1NP; a marker of bone formation) were shown in the restricted condition only [78]. Energy availability per se was not quantified and no measures were taken to prevent the reduction in micronutrient availability associated with energy restriction, which will have influenced the data. It has also been shown that P1NP concentrations are significantly reduced in active women following five days at an energy availability of 15kcal·kgLBM^−1^·day^−1^; *p* = 0.01, *d* = 0.36) when compared to the same duration at 45kcal·kgLBM^−1^·day^−1^ [10]. A significant effect was not shown in active men following the same protocol (*p* = 0.12, *d* = 0.31), although the effect size was small to moderate in both sexes [10]. In active women, P1NP was reduced after just three days at an energy availability of 15kcal·kgLBM^−1^·day^−1^ (*p* = 0.052, *d* = 0.36) when compared to the same duration at 45kcal·kgLBM^−1^·day^−1^ [79]. These studies by Papageorgiou et al. [10, 79] provided a multivitamin multi-mineral supplement to the restricted groups to maintain micronutrient availability. Findings suggest that both males and females may be affected but females were more sensitive. In support of this, lower levels of P1CP (a formation marker similar to P1NP) were evident in women following five days at a greater energy availability of 30kcal·kgLBM^−1^·day^−1^, compared to five days at an energy availability of 45kcal·kgLBM^−1^·day^−1^ [80].

Zanker and Swaine [78] showed no significant changes in two urine borne markers of bone resorption in response to 50% energy restriction compared with energy balance in male distance runners. However, energy availability was not reported and differences in micronutrient availability between conditions may have confounded the data. Papageorgiou et al. [10] also showed no effect of five days at an energy availability of 15kcal·kgLBM^−1^·day^−1^ on serum β-carboxyl-terminal cross-linked telopeptide of type 1 collagen (β-CTX; a marker of bone resorption) in active men. In active women, β-CTX significantly increased following five days at an energy availability of 15kcal·kgLBM^−1^·day^−1^ compared to 45kcal·kgLBM^−1^·day^−1^ [10], but not following three days at an energy availability of 15kcal·kgLBM^−1^·day^−1^ [79]. Furthermore, a urinary marker of bone resorption significantly decreased following five days only at the most severe level of LEA (10kcal·kgLBM^−1^·day^−1^) in women (*p* < 0.001, *d* = 0.96); but did not following more moderate levels of energy availability: 20 (*p* > 0.05, *d* = 0.16) or 30kcal·kgLBM^−1^·day^−1^ (*p* > 0.05, *d* = 0.16) [80]. Many different (re)modeling markers exist and heterogeneity between some of the studies makes direct comparisons difficult; however, the findings are consistent with those based on P1NP and β-CTX, which are the recommended international reference markers [77].

These findings show that LEA suppresses markers of bone formation within five days in active females and male WBEA. Females appear to respond with greater sensitivity than males, which suggests that markers of bone formation may also be suppressed in female WBEA in response to LEA, at least to a similar extent as in male WBEA. In male WBEA, it is possible that markers of bone resorption may only increase if LEA persists for longer than five days. Conversely, markers of bone resorption are increased in response to LEA in active females within five days, but only following LEA of greater duration or severity than is required to suppress markers of bone formation. This implies bone formation is more acutely affected by LEA than bone resorption which may lead to net bone breakdown. Due to a lack of evidence, it remains unclear whether the effect on bone resorption is the same in female athletes versus physically active women. Furthermore, in all studies that restrict energy availability, the availability of at least one of the macronutrients (carbohydrate, protein or fat) is inevitably reduced in the restricted condition and it has been shown that β-CTX is increased independent of energy availability in situations of reduced carbohydrate availability [81]. The contribution of reduced macronutrient availability to the effects described could not be determined.

The relationships between acute changes in markers of bone formation and resorption in response to LEA and long-term bone health are unclear. Sustained and localized acceleration of bone remodeling may play a role in the pathogenesis of bone stress injury in athletes [82, 83]. However, bone (re)modeling markers are typically measured systemically and, therefore, any change in the balance of such markers does not necessarily reflect a change in bone remodeling at a specific site. Accordingly, prospective research has shown no significant differences in bone (re)modeling markers between athletes and military recruits who suffered a stress fracture and those who did not [84, 85]. It is commonly reported that a reduction in bone resorption leads to long-term bone accrual; however, in an individual bone remodeling unit, resorptive osteoclast cells initiate the remodeling cycle and precede bone formation [86]. Thus, suppression of resorption might actually inhibit adaptation [87]. Although the acute effects of LEA on bone (re)modeling markers cannot yet be interpreted in terms of long-term bone health, it is reasonable to assume that preventing the acute effects from occurring would be beneficial given that evidence suggests the long-term effect of LEA is detrimental to bone structure, strength and stress injury risk.

That markers of bone (re)modeling respond acutely to LEA offers an exciting avenue to explore whether high-impact interventions modify the bone’s response to LEA, without the methodological challenge of controlling or monitoring diet and exercise for several months (at least). However, the bone (re)modeling response to repeated days of high-impact exercise is poorly understood. The existing data show either no change or a reduction in markers of bone formation and resorption [88, 89], and none of the previous research has simultaneously manipulated energy availability. Also, it is not clear to what degree these findings are due to the interventions per se, or merely a result of biological variation due to poor standardization [86]. One study was conducted in a cohort of hospitalized anorexia nervosa patients, which provides little information regarding whether high-impact exercise might prevent the acute effects of LEA in active populations [89]. Furthermore, the markers measured in response to short-term exercise interventions are often different from those measured in response to short-term LEA, which limits direct comparisons.

Using prolonged moderate impact running exercise, Papageorgiou et al. [79] exposed young active women to three conditions (each 3 days in duration) in a randomized crossover design: LEA (15kcal·kgLBM^−1^·day^−1^) with daily exercise, equally LEA non-exercise, and balanced energy availability (45kcal·kgLBM^−1^·day^−1^) with no exercise. P1NP was significantly reduced in the LEA non-exercise condition (*p* < 0.001, *d* = 0.91) but not in the LEA daily exercise condition (*p* = 0.14, *d* = 0.30). There was no effect on β-CTX in any condition [79]. It was suggested that daily moderate impact exercise might have prevented the acute effects of LEA on bone formation in active individuals. However, it is not known whether this effect persists over a longer period or whether such effects translate to WBEA who are more accustomed to the mechanical loading associated with daily prolonged running. As discussed, the effects of LEA on bone (re)modeling markers were more marked after five days than after three, but the independent effect of impact exercise has not been tested beyond a period of three days. Also, the intervention was neither time nor energy efficient (duration and energy expenditure were 129 ± 10 min day^−1^ and 30kcal·kg^−1^·day^−1^) and has not yet been tested in men [79]. Future research could use a similar design to that used by Papageorgiou et al. [79], but preferably use a longer duration of LEA or mimic a typical athlete training week to determine whether low repetition high-impact interventions prevent the acute effects of LEA on markers of bone (re)modeling in male and female WBEA. Findings may be used to improve evidence-informed practice, and inform longitudinal studies designed to investigate the effect of such interventions on long-term bone health and stress injury in athletes at risk of LEA.

## Conclusion

WBEA are frequently exposed to episodes of LEA in association with the demands of their sport. In female athletes, including WBEA, LEA is identified as a causative factor in FHA which, in turn, is associated with impaired bone health, including lower BMD, bone mineral content, and trabecular volumetric BMD, thinner cortices, and reduced bone strength. These impairments have been observed at load bearing sites, such as the proximal femur and tibia, and may increase the risk of bone stress injury as well as osteoporotic fracture. Not until recently was the existence of LEA associated bone loss acknowledged in men and, therefore, there are scarce data in male WBEA. High-impact exercise can be highly osteogenic with a low energy cost. Low repetition high-impact interventions have been shown to increase BMD, cortical thickness and estimated strength and preliminary evidence suggests that some of these effects may occur despite LEA. Such interventions may help attenuate bone change in WBEA and reduce the risk of bone stress injury in those at risk of LEA. Research examining the short-term effects of LEA in active men and women suggests that circulating markers of bone formation may be suppressed and circulating markers of bone resorption may be increased within five days, with women possibly responding to LEA with greater sensitivity. Prolonged moderate impact exercise may help mitigate the effects of short-term LEA; however, it is currently unclear whether this would be of benefit to long-term bone health. Given that bone health in WBEA can become compromised due to LEA, investigation of methods which may protect bone health in the face of LEA is of clinical importance.

## Footnotes

1 Lean body mass and fat-free mass are different measures of body composition that have been used interchangeably to normalise energy availability. As this is unlikely to have a major effect in the context of the current review, lean body mass has been reported throughout.
